# Reference values for skeletal muscle mass and fat mass measured by bioelectrical impedance in 390 565 UK adults

**DOI:** 10.1002/jcsm.12523

**Published:** 2020-01-13

**Authors:** Mei‐Man Lee, Susan A. Jebb, Jason Oke, Carmen Piernas

**Affiliations:** ^1^ Nuffield Department of Primary Care Health Sciences University of Oxford Oxford UK; ^2^ The George Institute for Global Health University of Oxford Oxford UK

**Keywords:** Skeletal muscle mass, Fat mass, Bioelectrical impedance analysis, Reference values

## Abstract

**Background:**

Loss of skeletal muscle mass (SMM) increases the risk of frailty and, together with excess fat mass (FM), is a risk factor for cardio‐metabolic disease. However, use of body composition measurements in nutritional surveillance and routine clinical practice is limited by the lack of reference data. Our aim was to produce age‐specific and sex‐specific reference values for SMM and FM in the White ethnic adult population in the UK. Secondary objectives were to examine the tracking over time using a subsample of the population with repeated measures of body composition and to assess the validity of these reference values in different ethnic subgroups.

**Methods:**

We used data from segmental bioelectrical impedance analysis (BIA) in 390 565 participants, aged 40–69 years, in the UK Biobank, and data from dual‐energy X‐ray absorptiometry from *n* = 905 participants to validate the BIA measurements. SMM was calculated as the sum of the predicted muscle mass from the limbs. The LMS method was used to produce percentile curves for the SMM index (SMMI = SMM/height^2^) and the FM index (FMI = FM/height^2^). We investigated the validity of the White ethnic reference values by plotting *z*‐scores (99.7% confidence interval) from Black and Asian groups to check if their confidence interval included zero. Longitudinal trajectories were predicted based on the baseline *z*‐scores and the correlation between repeated measurements at follow‐up.

**Results:**

The percentile curves show that SMMI declines in men from the age of 40, whereas in women, SMMI is more stable and decreases only slightly among women in the higher percentiles. FMI increases with age in both men and women. Women have higher FMI and lower SMMI than men in all age groups. The validity of the White‐based reference values for non‐White ethnic groups is poor. Longitudinal trajectories in body composition in the subsample of participants with a follow‐up assessment show regression towards the mean in both men and women, with some evidence of declining SMMI only among men. The predicted 90% limits for the expected 5 year trajectories of SMMI and FMI can be used to identify people with unusual trajectories and in clinical practice to identify and track individuals at risk of excessive loss of SMM.

**Conclusions:**

These body composition reference values developed from BIA in a middle/older‐aged healthy White ethnic population in the UK could be used as a simple assessment tool for nutritional surveillance and to identify individuals with low SMMI or high FMI who may be at increased risk of disease and/or frailty.

## Introduction

Loss of skeletal muscle mass (SMM), known as sarcopenia, carries an increased risk for poor health outcomes including frailty, increased morbidity, and premature mortality.^1^, ^2^, ^3^, ^4^ SMM is an important tissue for the maintenance of glucose homeostasis and is a biomarker for metabolic health.^3^, ^5^ On the contrary, fat mass (FM) has been shown to be strongly associated with increased risk of non‐communicable diseases, including hypertension, hyperlipidaemia, and insulin resistance.^6^ Sarcopenia in combination with excess body fat, known as sarcopenic obesity, is increasingly recognized as a major health concern in the aging population because of its association with a higher risk of cardio‐metabolic abnormalities.^2^, ^7^ The reported prevalence of sarcopenia varies from 10% to 50% in different populations, generally increasing with age to >50% among those aged ≥80 years old, with even higher prevalence among people who are resident in nursing homes.^8^ Sarcopenia and sarcopenic obesity remain inconsistently diagnosed due to a lack of consensus for measurement and definition.^9^ The development of sarcopenia in an aging population may be masked by weight stability, with reductions in muscle mass counterbalanced by increases in FM.^10^ Therefore, it is critical to develop standardized approaches to characterize muscle mass depletion, alone or in combination with increased adiposity,^11^ and to improve the clinical assessment of the main associated parameters of sarcopenia in the population.

Evidence suggests that body composition measurements may be more precise predictors of health than simpler measures of body weight because they account for differences in the proportion of fat and fat‐free mass (FFM).^12^ However, it is a challenge to make accurate measurements of body composition in routine clinical practice, and the practical considerations are even greater in people with reduced mobility. Bioelectrical impedance analysis (BIA) is an inexpensive and non‐invasive technique to assess body composition, which provides measures of FM and FFM with little or no more practical complexity than basic measurements of body weight. In addition, segmental BIA can provide measures of the composition of the limbs and trunk separately so that the fraction of muscle mass from the limbs, known as appendicular SMM, can be used as a proxy for SMM.^13^ Appendicular SMM accounts for approximately 75% of whole body SMM in adults and is the most modifiable fraction of whole body SMM.^14^ Comparisons of SMM from segmental BIA against reference methods to measure body composition such as dual‐energy X‐ray absorptiometry (DEXA) suggest that this is a reliable method to measure SMM, including in older populations.^15^, ^16^, ^17^, ^18^ Although FFM and SMM are highly correlated, increases in the proportion of connective tissue within the overall fat‐free tissue mass with aging and with increasing adiposity^11^ may explain why SMM appears to be a stronger biomarker for metabolic health than FFM.^16^, ^19^

The use of SMM and FM measurements in routine health surveillance and in clinical practice is currently limited by the lack of reference data to allow the classification of individuals at risk. The primary aim of this research is to construct reference values for body composition based on measurements made by bioelectrical impedance in a large healthy White ethnic population in the UK. Secondary aims include to assess the validity of these reference values in non‐White ethnic groups, and on a subsample of the population with repeated measures of body composition, to construct SMM and FM longitudinal trajectories to explore the tracking over time.

## Methods

### Design and study population

We used data from the UK Biobank study, a prospective population‐based cohort study comprising a baseline sample of 502 682 people aged between 40 and 69 years in 2006–2010 from across the UK. Participants included in the UK Biobank completed a full baseline assessment, including information on well‐being, lifestyle and behaviour, and medical history, and had a range of physical measurements and biological samples. A follow‐up assessment collecting the same measures was carried out in approximately 20 000 participants between 2012 and 2013.

In this analysis, we included 390 565 participants (*n* = 375, 512 Whites; *n* = 6283 Black; *n* = 8770 Asian) with complete data on body composition and all the relevant variables at baseline. We excluded participants who reported health conditions potentially affecting body composition,^20^ including type 1 diabetes (*n* = 4749); pregnancy (*n* = 330); cancer in last 5 years (*n* = 17 773); respiratory diseases (*n* = 12 538); self‐reported poor health (*n* = 15 093); more than one fall in last year (*n* = 22 779); renal failure (*n* = 461); endocrine disease (*n* = 3767); musculoskeletal disorders (*n* = 12 434); and HIV, cirrhosis, or other diseases (*n* = 1041). We also excluded any other ethnicities not covered above (*n* = 7185).

From the follow‐up sample, we selected *n* = 12 414 participants who had a repeated measurement of body composition, of White ethnic background, and free of the specified diseases at baseline and follow‐up. The follow‐up interval varies from 2 to 7 years, with 3, 4, and 5 years being the most frequent intervals.

The North West Multi‐centre Research Ethics Committee approved the study, and participants provided informed consent to take part in the UK Biobank. Detailed information about the study can be found at http://www.ukbiobank.ac.uk.

### Body composition measurements

Body weight (kg) and segmental single‐frequency BIA measurements were taken using a Tanita BC‐418 MA (Tanita Corporation, Arlington Heights, IL) in bare‐footed participants wearing light clothing. Body composition measures were recorded for the whole body and limbs separately, including measures of FM (kg), FFM (kg), predicted muscle mass (kg), body water (kg), and bone (kg). Segmental BIA provides measures of the composition of limbs and trunk separately; therefore, the sum of predicted muscle mass (kg) from the limbs, known as appendicular SMM, was calculated and used as a proxy for SMM. Standing height was measured using a Seca 202 scale (Seca, Hamburg, Germany) after removal of shoes. Both SMM and FM were normalized for height by dividing by height^2^ [SMM index (SMMI) and FM index (FMI)]. Using measures of SMM and FM normalized for height can account for the fact that people with the same body weight and FM percentage who differ in height will have different body composition status, or in other words, a higher FMI will result from a shorter person compared with a taller person with the same body weight and FM percentage. For example, two people of 1.5 and 1.8 m of height with 100 kg from which 40 kg is FM would both have 40% FM. However, the first person would be overweight with an elevated FMI compared with the second person.^11^

We examined the validity of the BIA measurements against DEXA as the reference method using the correlation and agreement of body composition measurements obtained from each method in a subsample of participants (*n* = 905) (see Supporting Information, Appendix S1).

### Statistical analysis

- Development of cross‐sectional reference values for SMMI and FMI in a White ethnic group

For the White ethnic group, we generated smoothed percentile curves for SMMI and FMI using generalized additive models (R package *gamlss*).^21^ The models are extensions of the LMS model that summarizes the data in terms of three smooth age‐specific curves, namely, L (*lambda*), M (*mu*), and S (*sigma*).^22^, ^23^, ^24^ This method does not assume that the outcome follows a normal distribution but instead employs a more flexible distribution that can accommodate skewed or kurtotic data. Parameters corresponding to centrality, dispersion, skewness, and kurtosis are first estimated using maximum likelihood methods for each time point, and for each parameter in turn, trends are smoothed using non‐parametric regression. Percentiles are then found by inverting the distribution function. We generated sex‐specific SMMI‐ and FMI‐for‐age percentiles curves and reference values for the 2nd, 9th, 25th, 50th, 75th, 91st, and 98th percentiles that correspond approximately to *z*‐scores spaced at two‐thirds of a standard deviation (SD). Accordingly, each individual had a *z*‐score that corresponds to their position on the reference curves.

- Comparison of reference values with Asian and Black ethnic groups

We compared body composition reference values developed for the White ethnic sample with body composition measurements taken from Black and Asian participants. We used the same model described earlier to calculate the *z*‐scores for SMMI or FMI for Black and Asian ethnic groups. Age‐specific and sex‐specific *z*‐scores for Black and Asian participants were summarized using means and confidence intervals. We hypothesized that if the distribution of body composition values in Black or Asian participants are similar to White ethnic populations, then the *z*‐score confidence interval would include zero. If the confidence interval does not include zero, then this would indicate population‐wide differences in body composition. To account for multiple testing, we used 99.7% confidence intervals.

- Longitudinal trajectories in body composition

We used the subsample of White ethnic participants with repeated measurements of body composition to explore the tracking of percentile position over time. First, we calculated the within‐subject coefficient of variation of SMMI and FMI in women and men. Second, we generated longitudinal trajectories based on the baseline *z*‐score and the correlation between repeated measurements of body composition.^25^ The correlation coefficient determines the amount of regression to the mean that occurs; if the correlation coefficient is close to or equal to 1, the trajectories will closely follow all of the cross‐sectional references curves, but when the correlation coefficient is less than 1, trajectories will be ‘regressed’ towards the mean or middle percentile value, with more regression occurring for curves further from the middle. We used a regression model to estimate the correlation between the *z*‐scores of the repeated measures of SMMI and FMI as function of age and follow‐up interval. More details of the methods used are given in Supporting Information, *Appendix S2*.

The correlation coefficient estimates were used to predict longitudinal trajectories of SMMI and FMI values at 5 year intervals for 2nd, 9th, 25th, 50th, 75th, 91st, and 98th percentiles starting at age 40 for men and women. Longitudinal trajectories are presented as graphs, with the 9th, 50th, and 91st cross‐sectional reference percentiles superimposed for context. We also calculated the 90% limits of the expected trajectory for SMMI and FMI for ages of 40, 50, and 60. This analysis will allow to classify people whose trajectories fall outside these upper and lower limits.^25^

## Results

The characteristics of the final sample can be found in Supporting Information, *Tables* *S1* and *S2*. For the White ethnic group, mean age (SD) was 56.6 (8.1) in men and 56.1 (8.0) in women, with approximately 24% of men and women in the 60‐ to 64‐year‐old age group. Mean SMMI (SD) was 8.75 (0.99) kg/m^2^ in men and 6.9 (0.78) kg/m^2^ in women. Mean FMI (SD) was 7.11 (2.52) kg/m^2^ in men and 9.97 (3.60) kg/m^2^ in women.

The comparison of measurements obtained from BIA vs. DEXA in a subsample of participants showed high correlations for both SMM and FM (intraclass correlation coefficient > 0.8, all *P* < 0.001). Bland–Altman plots showed very small differences and limits of agreement for SMM but slightly larger differences and wider limits for FM especially in men. BIA overestimated SMM by 2.5% in women and 1.9% in men but underestimated FM by 3% in women and 11% in men (Supporting Information, *Appendix S1*).

**Figure 1:**
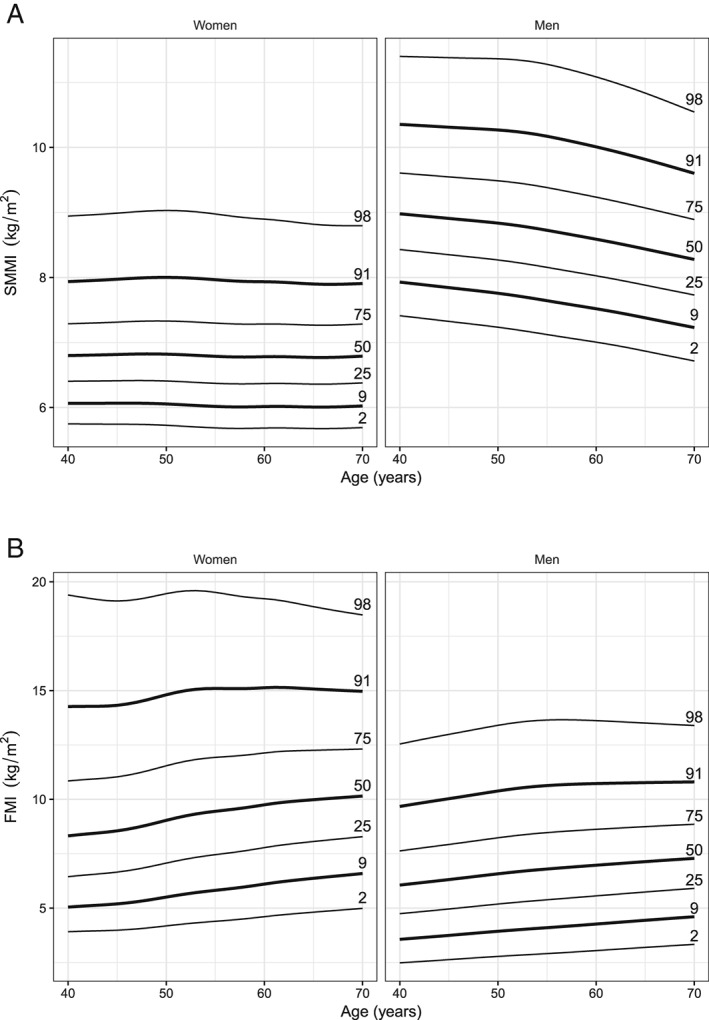
Skeletal muscle mass index (SMMI) (*A*) and fat mass index (FMI) (*B*) reference percentiles for men and women between ages 40 and 69 years in the White ethnic group.

*Figure* 1 shows the SMMI‐ and FMI‐for‐age percentile curves for men and women (the tabulated data can be found in Supporting Information, *Tables S3*–*S6*). The percentile curves show similarities in shape and variance between men and women; however, women have a higher FMI and lower SMMI compared with men in all age groups. At age 50, the median SMMI equates to 8.84 kg/m^2^ in men, compared with 6.82 kg/m^2^ in women. In men, the 50th percentile declines steadily with age from 8.98 to 8.31 kg/m^2^. SMMI is largely unchanged with age for most women with the 50th percentile between 6.80 and 6.78 kg/m^2^, except in women in higher percentiles, where SMMI declines slightly over 55 years. FMI increases with age in both women and men. Between ages 40 and 69 years, the 50th percentile increases from 8.32 to 10.12 kg/m^2^ in women and from 6.06 to 7.26 kg/m^2^ in men.

**Figure 2:**
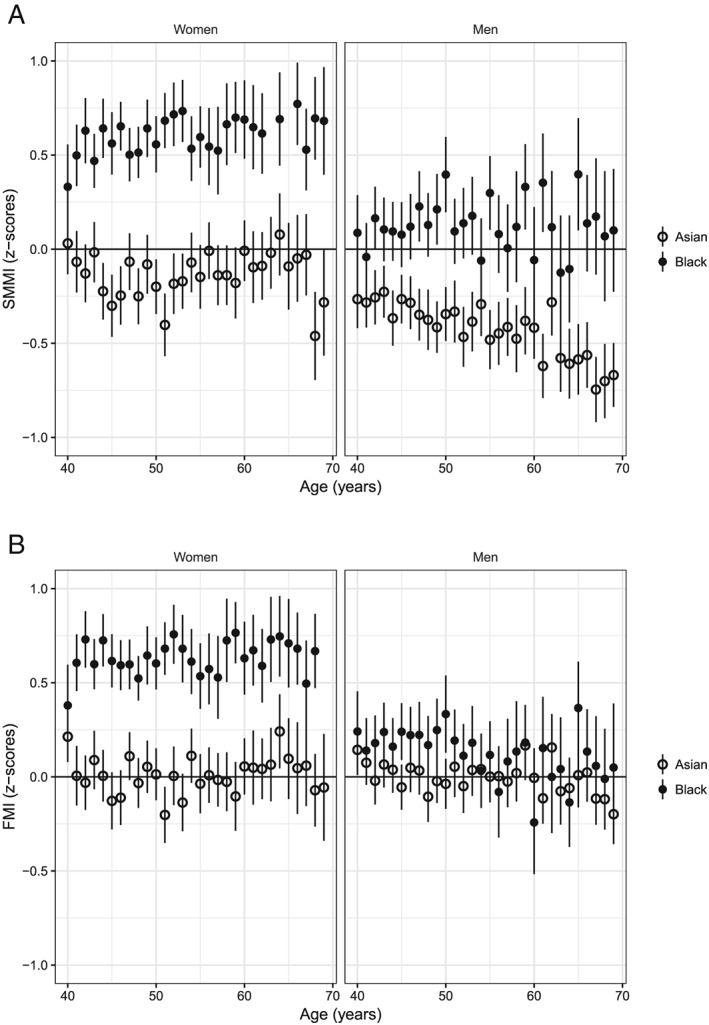
Comparisons between the White‐based reference values for skeletal muscle mass index (SMMI) (*A*) and fat mass index (FMI) (*B*) and the body composition measurements from the Asian and Black ethnic groups, showing means and confidence intervals for the *z*‐scores for each age.

*Figure* 2 shows the mean and 99.7% confidence interval of the *z*‐scores for SMMI and FMI in Asian and Black ethnic groups, stratified by sex and age. For Asian women, the *z*‐scores largely agreed with the White‐based reference values for FMI but were slightly lower for SMMI. For Asian men, the *z*‐scores agreed with the White‐based reference values for FMI but were too low for SMMI with the discrepancy increasing with age. For Black women, the White‐based reference resulted in higher *z*‐scores for both SMMI and FMI, and for Black men, the *z*‐scores varied greatly for both SMMI and FMI.

**Figure 3:**
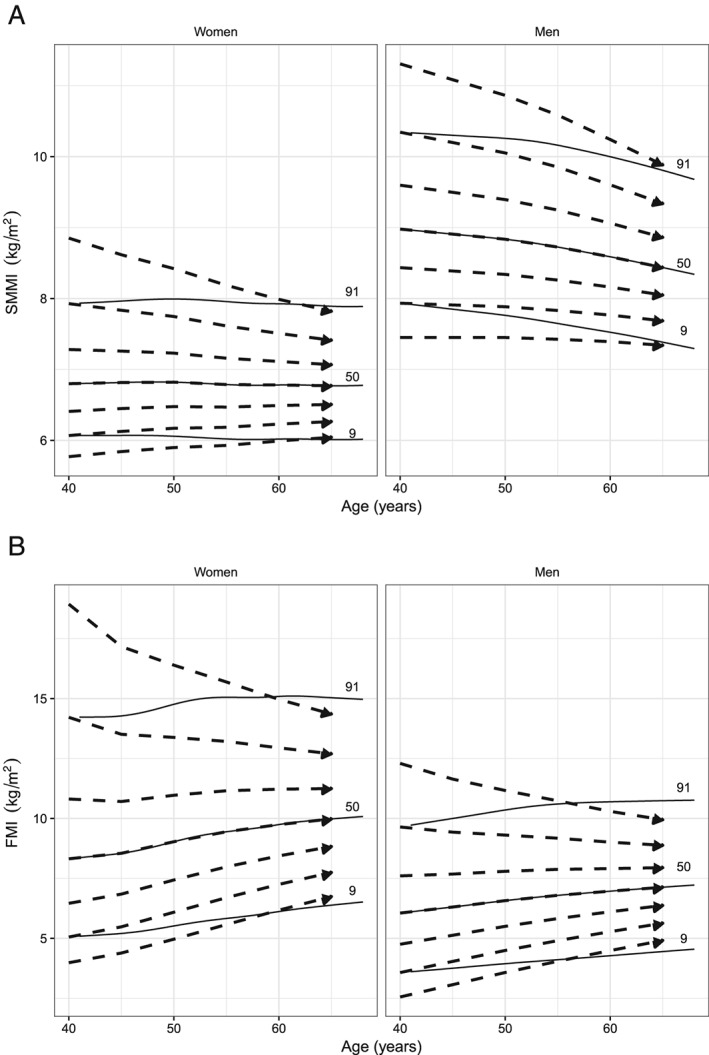
Longitudinal trajectories (dashed lines) of skeletal muscle mass index (SMMI) (*A*) and fat mass index (FMI) (*B*) values at 5 year intervals for 2nd, 9th, 25th, 50th, 75th, 91st, and 98th percentiles starting at age 40 for men and women, superimposed with the cross‐sectional percentiles (9th, 50th, and 91st) (solid lines).

Longitudinal trajectories in SMMI and FMI for the subsample of White ethnic participants with repeated body composition measurements are shown in *Figure* 3 (dashed lines), and the regression model of correlations of *z*‐scores between baseline and follow‐up is summarized in Supporting Information, *Tables S7* and *S8*. The predicted trajectories represent the median trajectories of individuals started at that percentiles position and measured every 5 years. The longitudinal trajectories coincide with the cross‐sectional percentiles (solid lines) at age 40 but thereafter regress towards the median, resulting in the convergence patterns of longitudinal trajectories in *Figure* 3. The coefficient of variation is higher for FMI (0.12 and 0.13 for women and men, respectively) than for SMMI (0.03 for both men and women). The overall correlation coefficient (regardless of the follow‐up intervals) between measurements is 0.9 for SMMI and FMI for men and women. The extent of the expected regression to the mean depends on this correlation between measurements. Declines in SMMI are evident among men and more clear among women above the 50th percentiles. For FMI, the longitudinal trajectories present a higher degree of regression towards the mean in both men and women.

*Table* 1 shows the 90% limits of the expected 5 year trajectories for SMMI and FMI. That is, there is a 10% chance of changing body composition to an extent that goes beyond these limits (5% below the lower limit and 5% above the upper limit). For example, a 50‐year‐old man with SMMI 8.01 kg/m^2^ is expected to have SMMI 7.95 kg/m^2^ at age 55, with the 90% limits between 7.50 and 8.44 kg/m^2^. Thus, this person might be considered to lose an unusual amount of muscle mass if his SMMI at age 55 is below 7.50 kg/m^2^ or to gain an unusual amount of muscle mass if above 8.44 kg/m^2^. Regardless of age and sex, higher SMMI or FMI at baseline will have wider 90% limits of the expected values at follow‐up. Also, because of the larger within‐subject variation in FMI than in SMMI, the 90% limits of expected body composition at the 5 year follow‐up are wider for FMI than for SMMI.

**Table 1:** The expected values with 90% limits of skeletal muscle mass index and fat mass index at the 5 year follow‐up for men and women, starting at four different values (corresponding to *z*‐scores −2, −1, 1, 2) of skeletal muscle mass index and fat mass index for ages 40, 50, and 60

	Age (years)	SMMI				FMI			
		Baseline	After 5 years			Baseline	After 5 years		
			5% lower limit	Expected	5% upper limit		5% lower limit	Expected	5% upper limit
Men	40	7.45	7.04	7.45	7.86	2.56	1.99	3.07	4.41
		8.18	7.72	8.14	8.59	4.15	3.21	4.57	6.13
		9.95	9.25	9.83	10.53	8.56	6.49	8.5	11.28
		11.31	10.28	11.08	12.14	12.3	8.77	11.64	15.53
	50	7.27	6.83	7.26	7.7	2.86	2.23	3.35	4.72
		8.01	7.5	7.95	8.44	4.56	3.56	4.96	6.53
		9.85	9.03	9.67	10.44	9.21	7.07	9.09	11.84
		11.27	10.07	10.94	12.11	13.14	9.48	12.36	16.16
	60	7.04	6.56	7.02	7.48	3.13	2.48	3.64	4.99
		7.77	7.22	7.69	8.2	4.91	3.91	5.3	6.8
		9.59	8.69	9.33	10.1	9.58	7.48	9.36	11.81
		10.99	9.64	10.5	11.62	13.37	9.83	12.41	15.69
Women	40	5.77	5.56	5.84	6.16	3.99	3.35	4.39	5.77
		6.23	5.95	6.28	6.66	5.71	4.67	6.13	8.04
		7.58	6.98	7.53	8.27	12.38	9.09	12.02	16.06
		8.85	7.77	8.62	9.9	18.94	12.81	17.17	23.24
	50	5.75	5.5	5.79	6.11	4.27	3.63	4.86	6.4
		6.23	5.9	6.24	6.63	6.26	5.23	6.86	8.86
		7.63	6.95	7.51	8.27	13.02	10.07	12.91	16.66
		8.94	7.76	8.61	9.87	19.05	13.79	17.81	23.08
	60	5.71	5.49	5.78	6.1	4.71	3.96	5.31	6.92
		6.19	5.89	6.23	6.62	6.93	5.76	7.45	9.37
		7.58	6.93	7.47	8.18	13.51	10.63	13.16	16.37
		8.81	7.7	8.49	9.59	18.88	14.04	17.47	21.76

FMI, fat mass index; SMMI, skeletal muscle mass index.

## Discussion

We present novel age‐specific and sex‐specific reference values and percentiles for SMM and FM in a large, healthy White ethnic population in the UK, which can be used to assess the relationship between body composition and health outcomes and as part of nutritional surveillance to identify people at risk. Longitudinal trajectories measured in a subsample provide preliminary evidence to identify people where changes over time fall outside the normal range. There were clear differences in reference curves for non‐White populations, and use of these data to assess body composition in other ethnic groups is not advised.

The main strengths of the study include the large sample size and applicability to the healthy middle/older‐aged population of White ethnicity in the UK. Although there are various reference values for FFM in the literature, SMM may be a more relevant marker for sarcopenia.^26^, ^27^, ^28^, ^29^, ^30^ For this study, we defined clear and evidence‐based criteria intended to exclude people with chronic diseases or conditions that may affect body composition,^20^ and we used the most robust method for creating the reference values.^24^ The reported 5 year trajectories in SMMI and FMI are a novel aspect of this work with potential to be applied in routine clinical practice or population surveillance. These reference values have been derived from BIA measurements, which is an inexpensive and feasible method to use in large epidemiological studies or routine clinical practice. The specific scales used in this study were previously validated against DEXA in a mixed population of children and adults, and body composition estimates were found to be more accurate than those obtained from previous BIA estimates.^31^ We also showed high correlations for both SMM and FM with very small differences and limits of agreement for SMM, while the differences in FM were similar to previous validation studies.^16^, ^18^, ^31^ There is evidence suggesting that BIA is less accurate at high BMI levels than some other methods of assessing body composition, which are used in specialist or research centres.^32^, ^33^ Because algorithms to estimate body composition by BIA vary, we would advise that these reference data are used in combination with a Tanita BC‐418 MA segmental body composition analyser. However, a few studies comparing different analysers from this manufacturer or others have reported only small differences in % body fat (e.g. equivalent to 0.3–0.8 units of difference in FMI),^34^, ^35^, ^36^ suggesting that a participant would likely fall into the same percentile regardless of the method used considering that for any age and sex there is always >1 unit of difference between percentiles 2 and 9 in FMI (where the smallest difference is reported). Whether this is also true for SMMI where the differences between percentiles 2 and 9 are smaller (~0.5 units) is not clear and requires further study.

Hydration status may also affect the accuracy of the BIA measurement, but the UK Biobank protocol did not specify any standard procedures to deal with some important determinants of hydration before the measurement (e.g. instructing participants not to eat or drink). Data from a comprehensive review suggest that overall BIA works best in healthy subjects with a stable water balance.^37^ However, we do not expect to see age‐related dehydration in this sample where the age range goes from 40 to 69 years at baseline when the BIA was performed. In addition, BIA is much less sensitive to the amount of fluid recently drunk than usually imagined because the truncal component only accounts for 10% of the total body impedance.^38^ Previous studies have successfully developed and used centile charts without applying strict procedures for measurement.^22^, ^23^, ^30^ The lack of pre‐measurement standardization used in UK Biobank reflects the likely scenario in routine applications of this method.

Our results show that SMM declines in men from the age of 40, whereas in women, SMM is more stable and decreases only slightly among women in the higher percentiles. Similar patterns have been observed in a previous study that reported SMM reference values in a healthy White population in the USA.^26^ To our knowledge, this is the first study to report reference values for SMM for a middle‐aged population in the UK using BIA measurements. Other studies reporting reference values for body composition have used FFM, which comprises all non‐fat tissues, and it is an indirect marker of SMM. Overall, these previous studies show a greater decline in FFM with age in men and women than observed in the present analysis of SMM.^26^, ^27^, ^28^, ^29^, ^30^ However, some of these studies had unclear exclusion criteria, wider age ranges, and much smaller sample sizes, meaning these previous studies are likely to be less representative than the population of the UK Biobank. Increases in FM among men and women are evident with aging in our study and consistent with the observed results from other White populations.^26^, ^27^, ^28^, ^29^, ^39^, ^40^

These reference values are especially relevant because it is increasingly recognized that body weight and BMI are less able to identify people at increased risk than more specific measures of body composition, which may be especially important in aging and diseased populations. These new reference values are potentially applicable in the clinical setting and/or in field surveys to classify sarcopenia, as well as for the evaluation of interventions aiming to improve the nutritional status of populations with specific needs.

The validity of these White‐based reference values for the non‐White ethnic groups was poor. The BIA prediction algorithm inbuilt in the device is mostly based on White populations,^41^, ^42^ and thus, it is not clear whether the differences observed are due to errors in the basic assessment of body composition by BIA or biological differences in body composition and/or changes with age.^43^, ^44^, ^45^ Our descriptive data showed that overall SMM and FM among Asians in the UK Biobank were similar to Whites, although previous studies have found that Asians tend to have lower SMM and FM levels,^46^. We did not assess fat distribution although Asian populations are generally observed to have a higher proportion of visceral FM, reflecting both genetic and environmental differences between populations.^46^, ^47^ Compared with Whites, Black ethnic population have a higher SMM and higher FM only among women, which is consistent with the literature.^26^ Descriptive trends in total body composition with age seen in our study are broadly consistent with previous results.^39^, ^40^, ^48^

Our analysis of longitudinal trajectories in body composition in the subsample of participants with a follow‐up assessment showed regression towards the mean, with some evidence of declining SMMI only among men. We have reported the predicted 90% limits for the expected 5 year trajectories of SMMI and FMI, which can be used to identify people with unusual trajectories and in clinical practice to identify and track individuals at risk of excessive loss of SMM, a risk factor for frailty and premature mortality. The differences in the cross‐sectional vs. longitudinal analyses make clear the error that may be introduced if cross‐sectional reference values, which do not account for the within‐person variability, are used to track individuals over time.^49^

In conclusion, this study presents age‐specific and gender‐specific percentile curves for SMM and FM scaled for height, which can be used as reference values for healthy White individuals aged 40–69 in the UK. Greater use of specific measures of muscle mass would aid the diagnosis of sarcopenia and help to better understand the underlying pathophysiology of frailty.^50^ It will also be relevant for tracking patients with specific conditions such as respiratory diseases, cardiovascular disease, or cancer, which are known to affect SMM. BIA is a practical tool to use in both large‐scale field studies and in routine health care settings. Given that it also measures body weight, it could replace existing measurements of weight alone to provide a better prognostic indicator than BMI. Identifying people at risk will help to target interventions to those at greatest need, improve patients' outcomes, and reduce costs to the health system.

## Conflict of interest

The authors declare no conflicts of interest with regard to this research.

## Supporting information


**Table S1**. Number of observations and descriptive statistics in the reference database by age and gender in the White‐ethnic group.
**Table S2**. Number of observations and descriptive statistics in the reference database by age and gender in the non‐white ethnic group.
**Table S3**. SMMI percentiles for White women.
**Table S4**. SMMI percentiles for White men.
**Table S5**. FMI percentiles for White women.
**Table S6**. FMI percentiles for White men.
**Table S7**. Regression of Fisher's Z of the correlation coefficients between the z‐scores of SMMI at different ages.
**Table S8**. Regression of Fisher's Z of the correlation coefficients between the z‐scores of FMI at different ages.
**Appendix S1**. Validation of segmental single‐frequency bioelectrical impedance analysis (Tanita BC‐418 MA) with Dual energy X‐ray absorptiometry in a subsample of the UK Biobank population.
**Appendix S2**. Modelling the expected trajectories in body composition.
