# Corrigendum

**DOI:** 10.1002/jcsm.12586

**Published:** 2020-06-15

In,^1^ the Acknowledgements section was incomplete. The correct acknowledgement should read as follows:

## References

[1] Lee M‐M , Jebb SA , Oke J , Piernas C . Reference values for skeletal muscle mass and fat mass measured by bioelectrical impedance in 390 565 UK adults. J Cachexia Sarcopenia Muscle 2020;11:487–496. 31943835 10.1002/jcsm.12523 PMC7113534

