# Supplementary material for: Reference values for skeletal muscle mass and fat mass measured by bioelectrical impedance in 390 565 UK adults
Source: J Cachexia Sarcopenia Muscle. 2020 Jan 13;11(2):487–96. doi: 10.1002/jcsm.12523 (PMC7113534; doi:10.1002/jcsm.12523)
Supplement: Supplementary file 1 — Table S1. Number of observations and descriptive statistics in the reference database by age and gender in the White‐ethnic group. Table S2. Number of observations and descriptive statistics in the reference database by age and gender in the non‐white ethnic group. Table S3. SMMI percentiles for White women. Table S4. SMMI percentiles for White men. Table S5. FMI percentiles for White women. Table S6. FMI percentiles for White men. Table S7. Regression of Fisher's Z of the correlation coefficients between the z‐scores of SMMI at different ages. Table S8. Regression of Fisher's Z of the correlation coefficients between the z‐scores of FMI at different ages. Appendix S1. Validation of segmental single‐frequency bioelectrical impedance analysis (Tanita BC‐418 MA) with Dual energy X‐ray absorptiometry in a subsample of the UK Biobank population. Appendix S2. Modelling the expected trajectories in body composition. [file JCSM-11-487-s001.docx]

# ELECTRONIC SUPPORTING INFORMATION

Contents

[Table S1. Number of observations and descriptive statistics in the reference database by age and gender in the White-ethnic group 2](#_Toc26527719)

[Table S2. Number of observations and descriptive statistics in the reference database by age and gender in the non-white ethnic group 3](#_Toc26527720)

[Table S3. SMMI percentiles for White women 4](#_Toc26527721)

[Table S4. SMMI percentiles for White men 5](#_Toc26527722)

[Table S5. FMI percentiles for White women 6](#_Toc26527723)

[Table S6. FMI percentiles for White men 7](#_Toc26527724)

[Table S7. Regression of Fisher's Z of the correlation coefficients between the z-scores of SMMI at different ages 8](#_Toc26527725)

[Table S8. Regression of Fisher's Z of the correlation coefficients between the z-scores of FMI at different ages. 9](#_Toc26527726)

[Appendix S1 – Validation of segmental single-frequency bioelectrical impedance analysis (Tanita BC-418 MA) with Dual energy X-ray absorptiometry in a subsample of the UK Biobank population 10](#_Toc26527727)

[Appendix S2 – Modelling the expected trajectories in body composition 13](#_Toc26527728)

### Table S1. Number of observations and descriptive statistics in the reference database by age and gender in the White-ethnic group

|  | | | | | | | |
| --- | --- | --- | --- | --- | --- | --- | --- |
|  | | | | **SMMI** | | **FMI** | |
| **Sex** | **Age (years)** | **N** | **%** | **Mean** | **SD** | **Mean** | **SD** |
| Women | 40-44 | 21303 | 10.5 | 6.9 | 0.8 | 9.1 | 3.7 |
|  | 45-49 | 28323 | 14.0 | 6.9 | 0.8 | 9.4 | 3.7 |
|  | 50-54 | 32765 | 16.2 | 6.9 | 0.8 | 9.9 | 3.7 |
|  | 55-59 | 37853 | 18.7 | 6.9 | 0.8 | 10.1 | 3.6 |
|  | 60-64 | 48737 | 24.1 | 6.9 | 0.8 | 10.3 | 3.5 |
|  | 65-69 | 33122 | 16.4 | 6.9 | 0.8 | 10.5 | 3.3 |
| Men | 40-44 | 18238 | 10.5 | 9.1 | 1.0 | 6.5 | 2.4 |
|  | 45-49 | 22537 | 13.0 | 9.0 | 1.0 | 6.7 | 2.5 |
|  | 50-54 | 25643 | 14.8 | 8.9 | 1.0 | 7.0 | 2.6 |
|  | 55-59 | 30934 | 17.8 | 8.8 | 1.0 | 7.2 | 2.6 |
|  | 60-64 | 42273 | 24.4 | 8.6 | 1.0 | 7.3 | 2.5 |
|  | 65-69 | 33784 | 19.5 | 8.5 | 0.9 | 7.5 | 2.4 |

| Black-ethnic group | | | | | | | |
| --- | --- | --- | --- | --- | --- | --- | --- |
|  | | | | **SMMI** | | **FMI** | |
| **Sex** | **Age (years)** | **N** | **%** | **Mean** | **SD** | **Mean** | **SD** |
| Women | 40-44 | 716 | 20.4 | 7.4 | 0.9 | 11.6 | 4.3 |
|  | 45-49 | 899 | 25.6 | 7.4 | 0.9 | 11.6 | 4.1 |
|  | 50-54 | 738 | 21.0 | 7.5 | 1.0 | 12.5 | 4.3 |
|  | 55-59 | 472 | 13.4 | 7.4 | 0.9 | 12.5 | 4.2 |
|  | 60-64 | 376 | 10.7 | 7.4 | 0.9 | 12.7 | 3.6 |
|  | 65-69 | 310 | 8.8 | 7.4 | 0.9 | 12.6 | 3.5 |
| Men | 40-44 | 657 | 23.7 | 9.1 | 1.0 | 6.9 | 2.4 |
|  | 45-49 | 698 | 25.2 | 9.1 | 1.1 | 7.3 | 2.5 |
|  | 50-54 | 537 | 19.4 | 9.1 | 1.1 | 7.4 | 2.7 |
|  | 55-59 | 353 | 12.7 | 9.0 | 1.0 | 7.3 | 2.4 |
|  | 60-64 | 266 | 9.6 | 8.7 | 1.1 | 7.2 | 2.5 |
|  | 65-69 | 261 | 9.4 | 8.7 | 1.1 | 7.8 | 2.6 |
| Asian-ethnic group | | | | | | | |
|  | | | | **SMMI** | | **FMI** | |
| **Sex** | **Age (years)** | **N** | **%** | **Mean** | **SD** | **Mean** | **SD** |
| Women | 40-44 | 812 | 19.2 | 6.9 | 0.8 | 9.3 | 3.5 |
|  | 45-49 | 867 | 20.5 | 6.8 | 0.7 | 9.2 | 3.3 |
|  | 50-54 | 833 | 19.7 | 6.8 | 0.8 | 9.6 | 3.5 |
|  | 55-59 | 747 | 17.7 | 6.8 | 0.8 | 9.9 | 3.3 |
|  | 60-64 | 590 | 13.9 | 6.9 | 0.8 | 10.6 | 3.4 |
|  | 65-69 | 383 | 9.1 | 6.8 | 0.7 | 10.5 | 3.3 |
| Men | 40-44 | 980 | 21.6 | 8.8 | 0.9 | 6.5 | 2.1 |
|  | 45-49 | 903 | 19.9 | 8.7 | 0.9 | 6.6 | 2.1 |
|  | 50-54 | 778 | 17.1 | 8.6 | 0.9 | 7.0 | 2.3 |
|  | 55-59 | 686 | 15.1 | 8.4 | 0.9 | 7.2 | 2.1 |
|  | 60-64 | 604 | 13.3 | 8.2 | 0.9 | 7.2 | 2.3 |
|  | 65-69 | 587 | 12.9 | 7.9 | 0.8 | 7.2 | 2.1 |

### Table S2. Number of observations and descriptive statistics in the reference database by age and gender in the non-white ethnic group

### Table *S*3. SMMI percentiles for White women

| Age | C2 | C9 | C25 | C50 | C75 | C91 | C98 |
| --- | --- | --- | --- | --- | --- | --- | --- |
| 40 | 5.75 | 6.06 | 6.40 | 6.80 | 7.29 | 7.94 | 8.94 |
| 41 | 5.75 | 6.06 | 6.40 | 6.80 | 7.29 | 7.94 | 8.95 |
| 42 | 5.75 | 6.06 | 6.41 | 6.80 | 7.30 | 7.95 | 8.96 |
| 43 | 5.75 | 6.06 | 6.41 | 6.81 | 7.30 | 7.96 | 8.97 |
| 44 | 5.74 | 6.07 | 6.41 | 6.81 | 7.31 | 7.96 | 8.98 |
| 45 | 5.74 | 6.07 | 6.41 | 6.82 | 7.31 | 7.97 | 8.99 |
| 46 | 5.74 | 6.07 | 6.41 | 6.82 | 7.32 | 7.98 | 9.00 |
| 47 | 5.74 | 6.07 | 6.41 | 6.82 | 7.33 | 7.99 | 9.01 |
| 48 | 5.74 | 6.06 | 6.41 | 6.82 | 7.33 | 8.00 | 9.02 |
| 49 | 5.73 | 6.06 | 6.41 | 6.82 | 7.33 | 8.00 | 9.03 |
| 50 | 5.73 | 6.05 | 6.41 | 6.82 | 7.33 | 8.00 | 9.03 |
| 51 | 5.72 | 6.05 | 6.40 | 6.82 | 7.33 | 8.00 | 9.03 |
| 52 | 5.71 | 6.04 | 6.39 | 6.81 | 7.32 | 7.99 | 9.02 |
| 53 | 5.70 | 6.03 | 6.39 | 6.80 | 7.31 | 7.99 | 9.01 |
| 54 | 5.69 | 6.02 | 6.38 | 6.79 | 7.31 | 7.98 | 8.99 |
| 55 | 5.69 | 6.02 | 6.37 | 6.79 | 7.30 | 7.97 | 8.98 |
| 56 | 5.68 | 6.01 | 6.37 | 6.78 | 7.29 | 7.95 | 8.96 |
| 57 | 5.68 | 6.01 | 6.36 | 6.78 | 7.28 | 7.95 | 8.94 |
| 58 | 5.68 | 6.01 | 6.36 | 6.78 | 7.28 | 7.94 | 8.92 |
| 59 | 5.68 | 6.01 | 6.37 | 6.78 | 7.28 | 7.94 | 8.91 |
| 60 | 5.68 | 6.01 | 6.37 | 6.78 | 7.28 | 7.93 | 8.90 |
| 61 | 5.69 | 6.02 | 6.37 | 6.78 | 7.28 | 7.93 | 8.89 |
| 62 | 5.69 | 6.02 | 6.37 | 6.78 | 7.28 | 7.92 | 8.87 |
| 63 | 5.68 | 6.01 | 6.37 | 6.78 | 7.28 | 7.92 | 8.85 |
| 64 | 5.68 | 6.01 | 6.36 | 6.77 | 7.27 | 7.91 | 8.83 |
| 65 | 5.68 | 6.01 | 6.36 | 6.77 | 7.27 | 7.90 | 8.82 |
| 66 | 5.68 | 6.01 | 6.36 | 6.77 | 7.26 | 7.89 | 8.81 |
| 67 | 5.68 | 6.01 | 6.36 | 6.77 | 7.27 | 7.89 | 8.80 |
| 68 | 5.68 | 6.01 | 6.37 | 6.78 | 7.27 | 7.90 | 8.80 |
| 69 | 5.69 | 6.02 | 6.37 | 6.78 | 7.28 | 7.90 | 8.80 |

### Table *S*4. SMMI percentiles for White men

| Age | C2 | C9 | C25 | C50 | C75 | C91 | C98 |
| --- | --- | --- | --- | --- | --- | --- | --- |
| 40 | 7.41 | 7.93 | 8.43 | 8.98 | 9.61 | 10.35 | 11.40 |
| 41 | 7.39 | 7.91 | 8.41 | 8.96 | 9.59 | 10.35 | 11.40 |
| 42 | 7.38 | 7.89 | 8.40 | 8.95 | 9.58 | 10.34 | 11.39 |
| 43 | 7.36 | 7.88 | 8.38 | 8.94 | 9.57 | 10.33 | 11.39 |
| 44 | 7.34 | 7.86 | 8.37 | 8.92 | 9.56 | 10.32 | 11.38 |
| 45 | 7.32 | 7.84 | 8.35 | 8.91 | 9.55 | 10.31 | 11.38 |
| 46 | 7.31 | 7.83 | 8.33 | 8.89 | 9.53 | 10.30 | 11.38 |
| 47 | 7.29 | 7.81 | 8.32 | 8.88 | 9.52 | 10.29 | 11.37 |
| 48 | 7.27 | 7.79 | 8.30 | 8.87 | 9.51 | 10.29 | 11.37 |
| 49 | 7.25 | 7.78 | 8.29 | 8.85 | 9.50 | 10.28 | 11.37 |
| 50 | 7.23 | 7.76 | 8.27 | 8.84 | 9.49 | 10.27 | 11.36 |
| 51 | 7.21 | 7.74 | 8.25 | 8.82 | 9.47 | 10.26 | 11.36 |
| 52 | 7.19 | 7.72 | 8.23 | 8.80 | 9.45 | 10.24 | 11.34 |
| 53 | 7.17 | 7.69 | 8.21 | 8.78 | 9.43 | 10.22 | 11.33 |
| 54 | 7.15 | 7.67 | 8.18 | 8.75 | 9.41 | 10.20 | 11.31 |
| 55 | 7.12 | 7.64 | 8.16 | 8.73 | 9.38 | 10.17 | 11.28 |
| 56 | 7.10 | 7.62 | 8.13 | 8.70 | 9.35 | 10.14 | 11.24 |
| 57 | 7.07 | 7.59 | 8.10 | 8.67 | 9.32 | 10.11 | 11.21 |
| 58 | 7.05 | 7.57 | 8.08 | 8.64 | 9.30 | 10.08 | 11.17 |
| 59 | 7.03 | 7.54 | 8.05 | 8.62 | 9.27 | 10.04 | 11.13 |
| 60 | 7.01 | 7.52 | 8.03 | 8.59 | 9.24 | 10.01 | 11.08 |
| 61 | 6.98 | 7.49 | 8.00 | 8.56 | 9.20 | 9.97 | 11.04 |
| 62 | 6.95 | 7.47 | 7.97 | 8.53 | 9.17 | 9.94 | 10.99 |
| 63 | 6.93 | 7.44 | 7.94 | 8.50 | 9.14 | 9.90 | 10.94 |
| 64 | 6.90 | 7.41 | 7.91 | 8.47 | 9.10 | 9.86 | 10.89 |
| 65 | 6.87 | 7.38 | 7.88 | 8.44 | 9.07 | 9.82 | 10.84 |
| 66 | 6.84 | 7.35 | 7.85 | 8.40 | 9.03 | 9.77 | 10.78 |
| 67 | 6.81 | 7.32 | 7.82 | 8.37 | 9.00 | 9.73 | 10.72 |
| 68 | 6.78 | 7.29 | 7.79 | 8.34 | 8.96 | 9.69 | 10.66 |
| 69 | 6.75 | 7.26 | 7.76 | 8.31 | 8.93 | 9.64 | 10.60 |

### Table *S*5. FMI percentiles for White women

| Age | C2 | C9 | C25 | C50 | C75 | C91 | C98 |
| --- | --- | --- | --- | --- | --- | --- | --- |
| 40 | 3.91 | 5.04 | 6.44 | 8.32 | 10.85 | 14.26 | 19.39 |
| 41 | 3.93 | 5.08 | 6.49 | 8.37 | 10.88 | 14.27 | 19.32 |
| 42 | 3.94 | 5.10 | 6.53 | 8.41 | 10.92 | 14.27 | 19.25 |
| 43 | 3.95 | 5.13 | 6.57 | 8.45 | 10.95 | 14.28 | 19.18 |
| 44 | 3.97 | 5.16 | 6.61 | 8.49 | 10.98 | 14.29 | 19.13 |
| 45 | 3.98 | 5.19 | 6.65 | 8.55 | 11.03 | 14.32 | 19.12 |
| 46 | 4.01 | 5.24 | 6.71 | 8.62 | 11.10 | 14.38 | 19.14 |
| 47 | 4.04 | 5.29 | 6.79 | 8.71 | 11.19 | 14.46 | 19.19 |
| 48 | 4.08 | 5.36 | 6.87 | 8.81 | 11.30 | 14.57 | 19.26 |
| 49 | 4.13 | 5.43 | 6.97 | 8.92 | 11.42 | 14.69 | 19.36 |
| 50 | 4.18 | 5.50 | 7.07 | 9.03 | 11.54 | 14.80 | 19.45 |
| 51 | 4.23 | 5.58 | 7.16 | 9.14 | 11.65 | 14.91 | 19.53 |
| 52 | 4.28 | 5.65 | 7.25 | 9.24 | 11.75 | 15.00 | 19.58 |
| 53 | 4.32 | 5.71 | 7.33 | 9.32 | 11.83 | 15.06 | 19.59 |
| 54 | 4.36 | 5.77 | 7.40 | 9.39 | 11.89 | 15.09 | 19.57 |
| 55 | 4.39 | 5.82 | 7.46 | 9.45 | 11.93 | 15.10 | 19.51 |
| 56 | 4.43 | 5.86 | 7.51 | 9.50 | 11.96 | 15.09 | 19.44 |
| 57 | 4.46 | 5.92 | 7.57 | 9.55 | 11.99 | 15.09 | 19.37 |
| 58 | 4.51 | 5.97 | 7.63 | 9.61 | 12.03 | 15.09 | 19.30 |
| 59 | 4.55 | 6.04 | 7.70 | 9.68 | 12.08 | 15.11 | 19.26 |
| 60 | 4.60 | 6.10 | 7.78 | 9.75 | 12.13 | 15.13 | 19.22 |
| 61 | 4.65 | 6.17 | 7.85 | 9.81 | 12.18 | 15.14 | 19.18 |
| 62 | 4.70 | 6.22 | 7.91 | 9.87 | 12.21 | 15.14 | 19.11 |
| 63 | 4.74 | 6.27 | 7.96 | 9.91 | 12.23 | 15.12 | 19.03 |
| 64 | 4.77 | 6.32 | 8.01 | 9.95 | 12.24 | 15.10 | 18.94 |
| 65 | 4.81 | 6.37 | 8.06 | 9.98 | 12.25 | 15.07 | 18.86 |
| 66 | 4.85 | 6.41 | 8.11 | 10.02 | 12.27 | 15.05 | 18.77 |
| 67 | 4.88 | 6.46 | 8.15 | 10.05 | 12.28 | 15.03 | 18.69 |
| 68 | 4.92 | 6.50 | 8.19 | 10.08 | 12.29 | 15.00 | 18.62 |
| 69 | 4.95 | 6.54 | 8.24 | 10.12 | 12.30 | 14.98 | 18.55 |

### Table *S*6. FMI percentiles for White men

| Age | C2 | C9 | C25 | C50 | C75 | C91 | C98 |
| --- | --- | --- | --- | --- | --- | --- | --- |
| 40 | 2.48 | 3.56 | 4.74 | 6.06 | 7.63 | 9.67 | 12.54 |
| 41 | 2.51 | 3.60 | 4.78 | 6.11 | 7.69 | 9.75 | 12.64 |
| 42 | 2.54 | 3.63 | 4.83 | 6.16 | 7.75 | 9.82 | 12.73 |
| 43 | 2.57 | 3.67 | 4.87 | 6.21 | 7.81 | 9.89 | 12.82 |
| 44 | 2.60 | 3.71 | 4.91 | 6.26 | 7.87 | 9.96 | 12.90 |
| 45 | 2.63 | 3.74 | 4.96 | 6.31 | 7.93 | 10.03 | 12.99 |
| 46 | 2.66 | 3.78 | 5.00 | 6.36 | 7.99 | 10.10 | 13.07 |
| 47 | 2.69 | 3.82 | 5.05 | 6.42 | 8.05 | 10.17 | 13.15 |
| 48 | 2.72 | 3.86 | 5.09 | 6.47 | 8.11 | 10.24 | 13.24 |
| 49 | 2.75 | 3.90 | 5.14 | 6.52 | 8.17 | 10.31 | 13.32 |
| 50 | 2.78 | 3.93 | 5.19 | 6.58 | 8.23 | 10.38 | 13.40 |
| 51 | 2.81 | 3.97 | 5.23 | 6.63 | 8.29 | 10.45 | 13.47 |
| 52 | 2.83 | 4.00 | 5.27 | 6.67 | 8.34 | 10.51 | 13.54 |
| 53 | 2.86 | 4.04 | 5.31 | 6.72 | 8.39 | 10.56 | 13.59 |
| 54 | 2.88 | 4.07 | 5.35 | 6.76 | 8.43 | 10.60 | 13.62 |
| 55 | 2.91 | 4.10 | 5.38 | 6.80 | 8.47 | 10.63 | 13.65 |
| 56 | 2.93 | 4.13 | 5.42 | 6.83 | 8.50 | 10.66 | 13.66 |
| 57 | 2.96 | 4.16 | 5.45 | 6.87 | 8.54 | 10.68 | 13.66 |
| 58 | 2.99 | 4.20 | 5.49 | 6.90 | 8.57 | 10.70 | 13.65 |
| 59 | 3.02 | 4.23 | 5.52 | 6.94 | 8.59 | 10.71 | 13.64 |
| 60 | 3.05 | 4.27 | 5.56 | 6.97 | 8.62 | 10.72 | 13.62 |
| 61 | 3.08 | 4.30 | 5.60 | 7.00 | 8.65 | 10.73 | 13.60 |
| 62 | 3.10 | 4.33 | 5.63 | 7.04 | 8.67 | 10.74 | 13.58 |
| 63 | 3.13 | 4.37 | 5.67 | 7.07 | 8.69 | 10.75 | 13.55 |
| 64 | 3.16 | 4.40 | 5.70 | 7.10 | 8.72 | 10.76 | 13.53 |
| 65 | 3.19 | 4.44 | 5.74 | 7.13 | 8.74 | 10.77 | 13.51 |
| 66 | 3.22 | 4.47 | 5.77 | 7.17 | 8.77 | 10.78 | 13.48 |
| 67 | 3.25 | 4.51 | 5.81 | 7.20 | 8.79 | 10.78 | 13.46 |
| 68 | 3.28 | 4.54 | 5.84 | 7.23 | 8.81 | 10.79 | 13.44 |
| 69 | 3.30 | 4.57 | 5.87 | 7.26 | 8.83 | 10.79 | 13.41 |

### Table S7. Regression of Fisher's Z of the correlation coefficients between the z-scores of SMMI at different ages

|  | | | | |
| --- | --- | --- | --- | --- |
|  | **Men** | | **Women** | |
| *Predictors* | *Estimates* | *p* | *Estimates* | *p* |
| intercept | 1.92 (1.57 – 2.27) | **<0.001** | 1.56 (1.25 – 1.87) | **<0.001** |
| mean age | -0.01 (-0.01 – -0.00) | **0.015** | -0.00 (-0.01 – 0.00) | 0.905 |
| age interval | 0.02 (-0.01 – 0.06) | 0.244 | -0.00 (-0.03 – 0.03) | 0.987 |

### Table S8. Regression of Fisher's Z of the correlation coefficients between the z-scores of FMI at different ages.

|  | | | | |
| --- | --- | --- | --- | --- |
|  | **Men** | | **Women** | |
| *Predictors* | *Estimates* | *p* | *Estimates* | *p* |
| intercept | 1.47 (1.04 – 1.91) | **<0.001** | 1.53 (1.18 – 1.89) | **<0.001** |
| mean age | 0.00 (-0.00 – 0.01) | 0.263 | 0.00 (-0.00 – 0.01) | 0.369 |
| age interval | -0.06 (-0.10 – -0.01) | **0.017** | -0.04 (-0.08 – -0.00) | **0.044** |

### Appendix S1 – Validation of segmental single-frequency bioelectrical impedance analysis (Tanita BC-418 MA) with Dual energy X-ray absorptiometry in a subsample of the UK Biobank population

**Methods**

***Study sample***

We used a subsample of UK Biobank participants n=905 (women n=433, men n=472) who were weight stable (within 3% of their initial body weight^[[1]](#footnote-1)^) due to the body composition measurements obtained by BIA and DEXA not taken at the same time. This subsample of participants only include participants of white ethnicity and free of health conditions which may affect body composition (details in the Methods section of the main manuscript).

***Body composition assessment***

a) Bioelectrical impedance analysis (BIA)

A Tanita BC-418 MA scale (Tanita Corporation, Arlington Heights, IL) was used in bare-footed participants wearing light clothing to obtain measurements of total body fat mass (FM, kg) and fat-free mass (FFM, kg) and predicted muscle mass from the limbs (kg). The sum of predicted muscle mass (kg) from the limbs was calculated to obtain the appendicular skeletal muscle mass (aSMM, kg).

The BIA measurements used in this validation study were taken as part of the follow-up re-measurement performed in 2012-2013.

b) Dual energy X-ray absorptiometry (DEXA)

A whole-body DEXA was performed with a GE-Lunar iDXA (GE Healthcare, Madison, Wisconsin, USA) with the subjects in supine position. The images were analysed using the GE encore software by the radiographer. Whole body fat mass (FM, kg), fat-free mass (FFM, kg) and aSMM (calculated as the sum of lean mass in each limb in kg) were used.

The DEXA measurements used in this validation study were taken as part of the imaging study performed in 2014-2015.

***Statistical analysis***

Data was analysed separately for men and women and presented as mean (SD). Intraclass correlation coefficients were used to assess the association between body composition measurements obtained from BIA and DEXA. Agreements and systematic differences between the methods were examined using Bland-Altman plot. All analyses were performed in Stata 14 with p<0.05 set to denote statistical significance.

***Results***

Body composition measurements by BIA and DEXA showed high correlations for both men and women (ICC>0.8, all *p*<0.001).

Agreements between the two methods were examined using the Bland-Altman plots. There were small differences and narrow limits of agreement for aSMM in both men and women, but somewhat slightly larger differences and wider limits for FFM and FM, especially in men.

Compared to DEXA, BIA overestimated aSMM and FFM by 2.5% and 3.6% respectively; while underestimating FM by 2.8% in women. In men, BIA overestimated aSMM and FFM by 1.9% and 6% respectively; while underestimating FM by 11%.

Table S9. Body composition measurements taken from BIA and DEXA by gender

|  | Women n=433 | | | | | Men n=472 | | | | |
| --- | --- | --- | --- | --- | --- | --- | --- | --- | --- | --- |
|  | DEXA | | BIA | | ICC* | DEXA | | BIA | | ICC* |
| All (n=905) | Mean | SD | Mean | SD |  | Mean | SD | Mean | SD |  |
| *aSMM kg* | 17.1 | 2.3 | 17.6 | 1.9 | 0.87 | 25.5 | 3.5 | 26.0 | 3.5 | 0.90 |
| *Total Body Fat kg* | 25.0 | 8.3 | 24.3 | 8.2 | 0.97 | 24.2 | 7.9 | 21.7 | 7.2 | 0.88 |
| *Total Fat Free Mass kg* | 41.5 | 4.5 | 43.0 | 4.1 | 0.85 | 58.4 | 6.6 | 62.0 | 7.2 | 0.80 |

* ICC intraclass correlation coefficient, all *p*<0.001


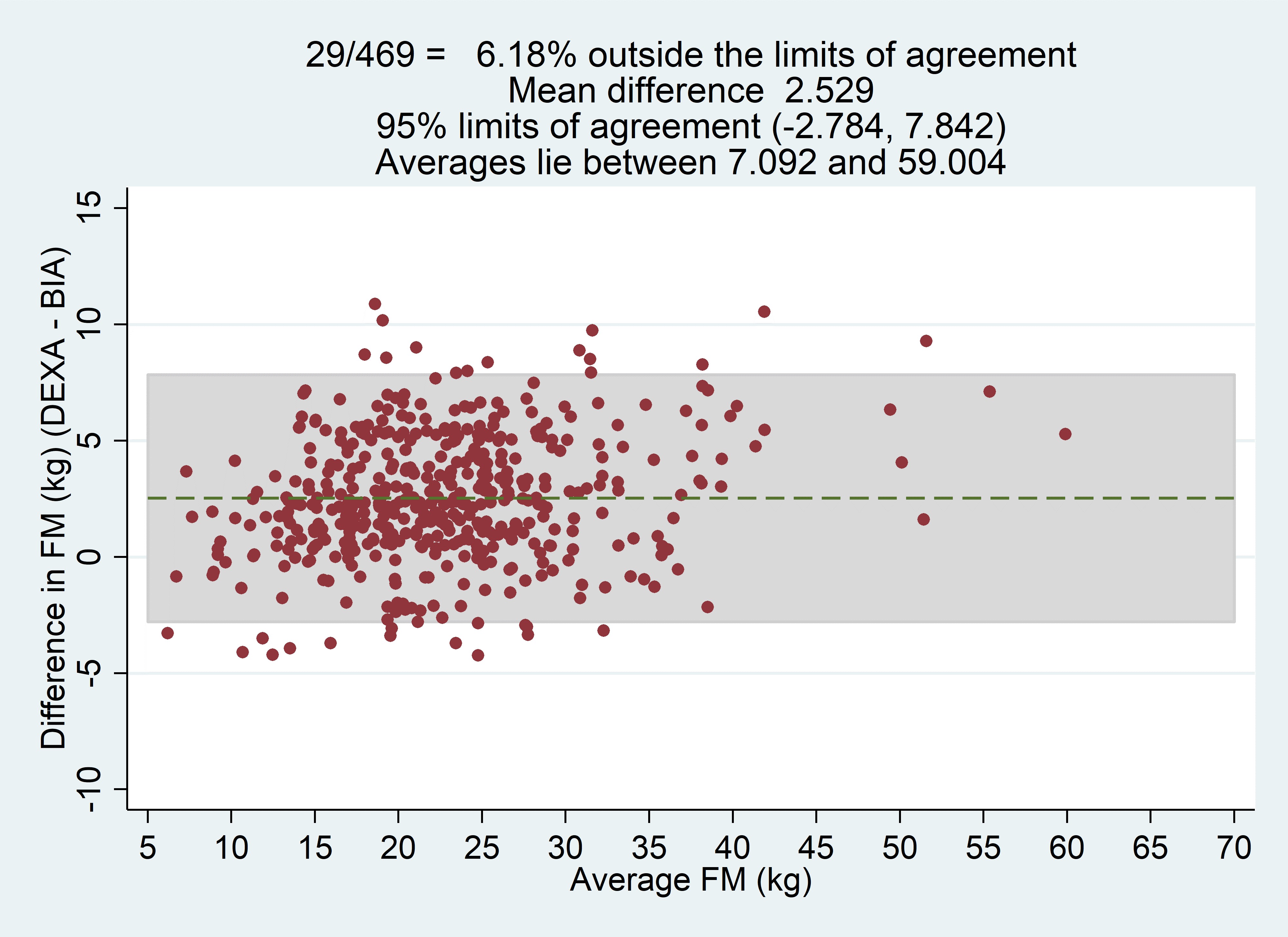

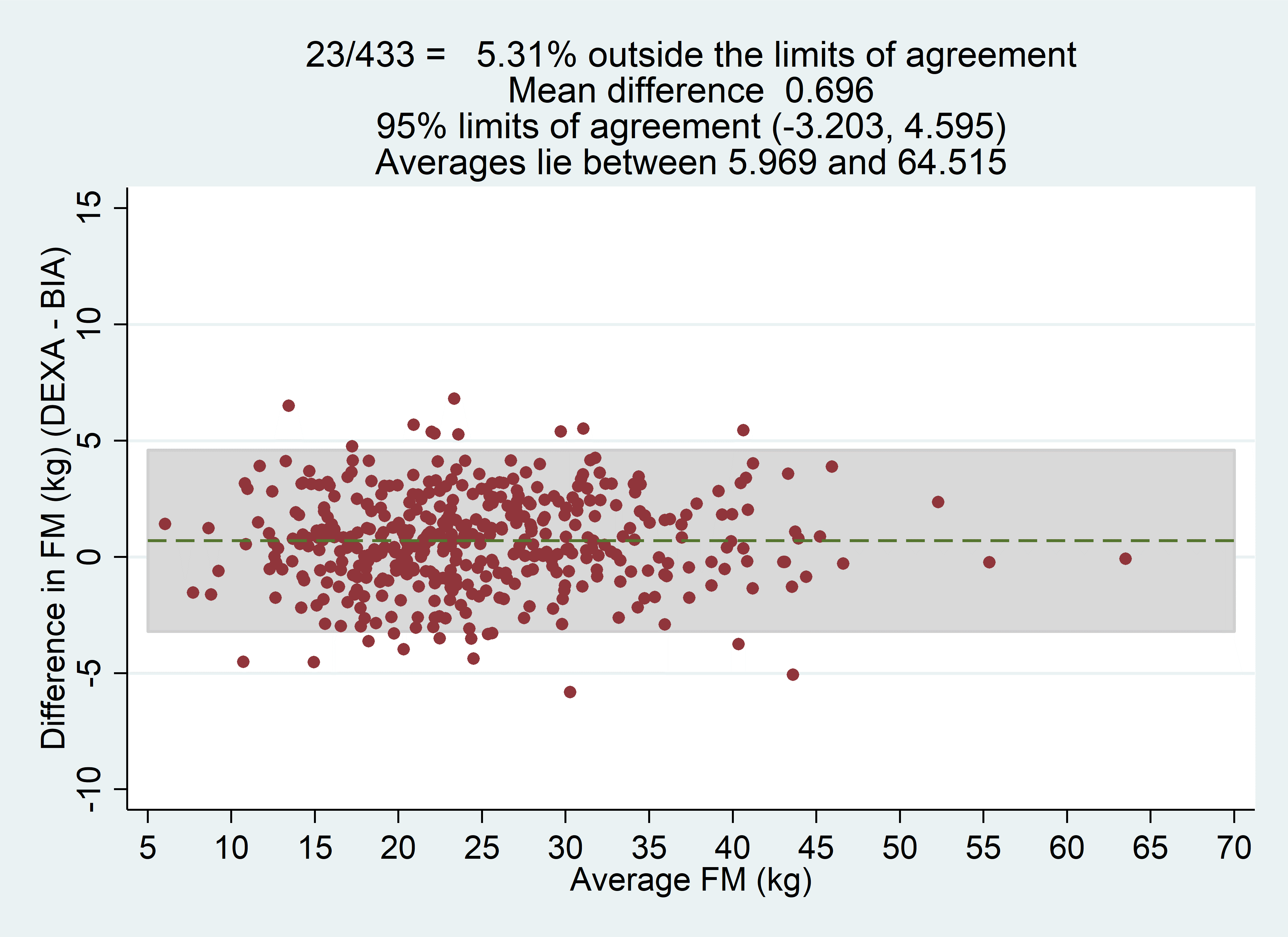

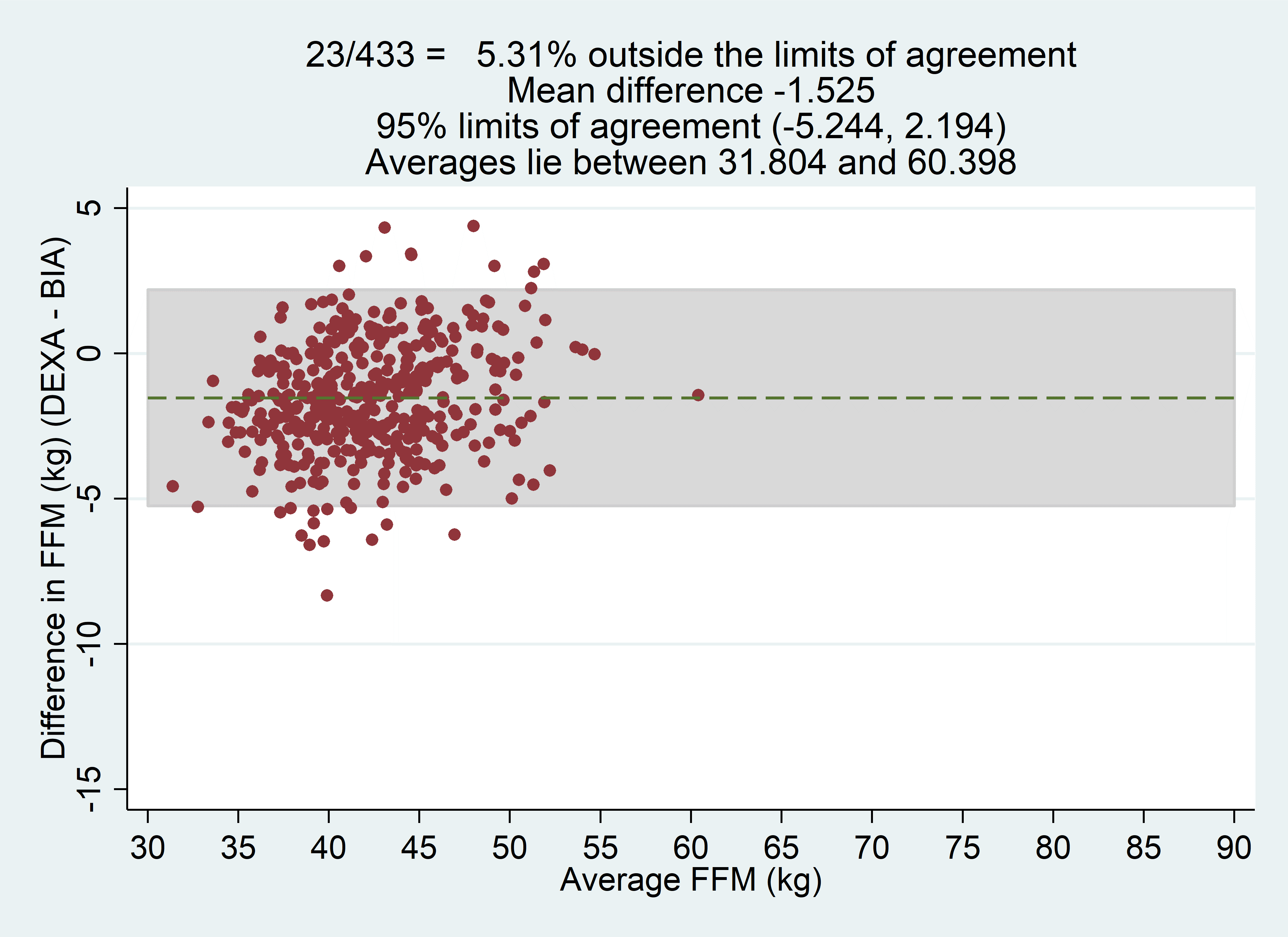

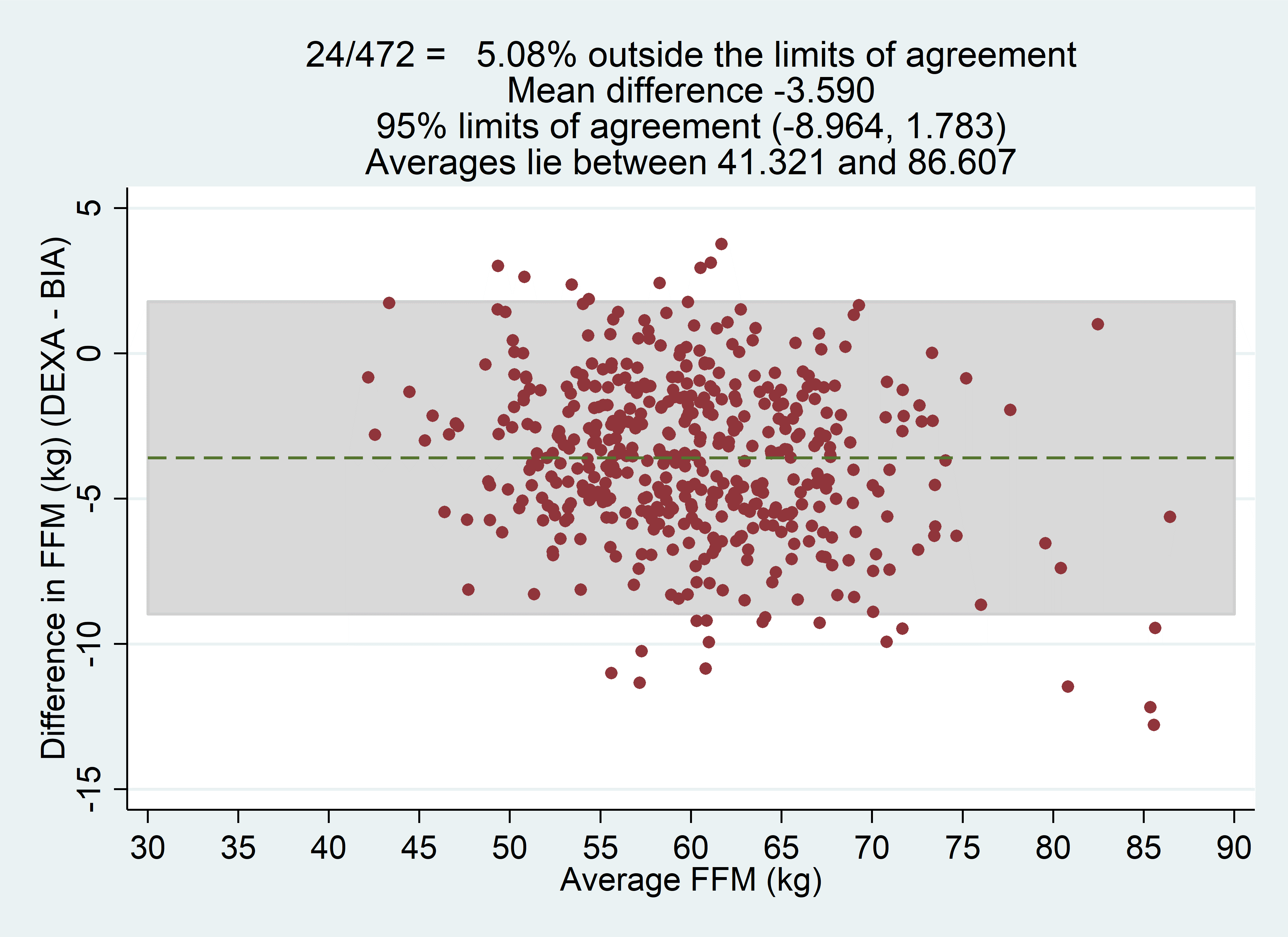

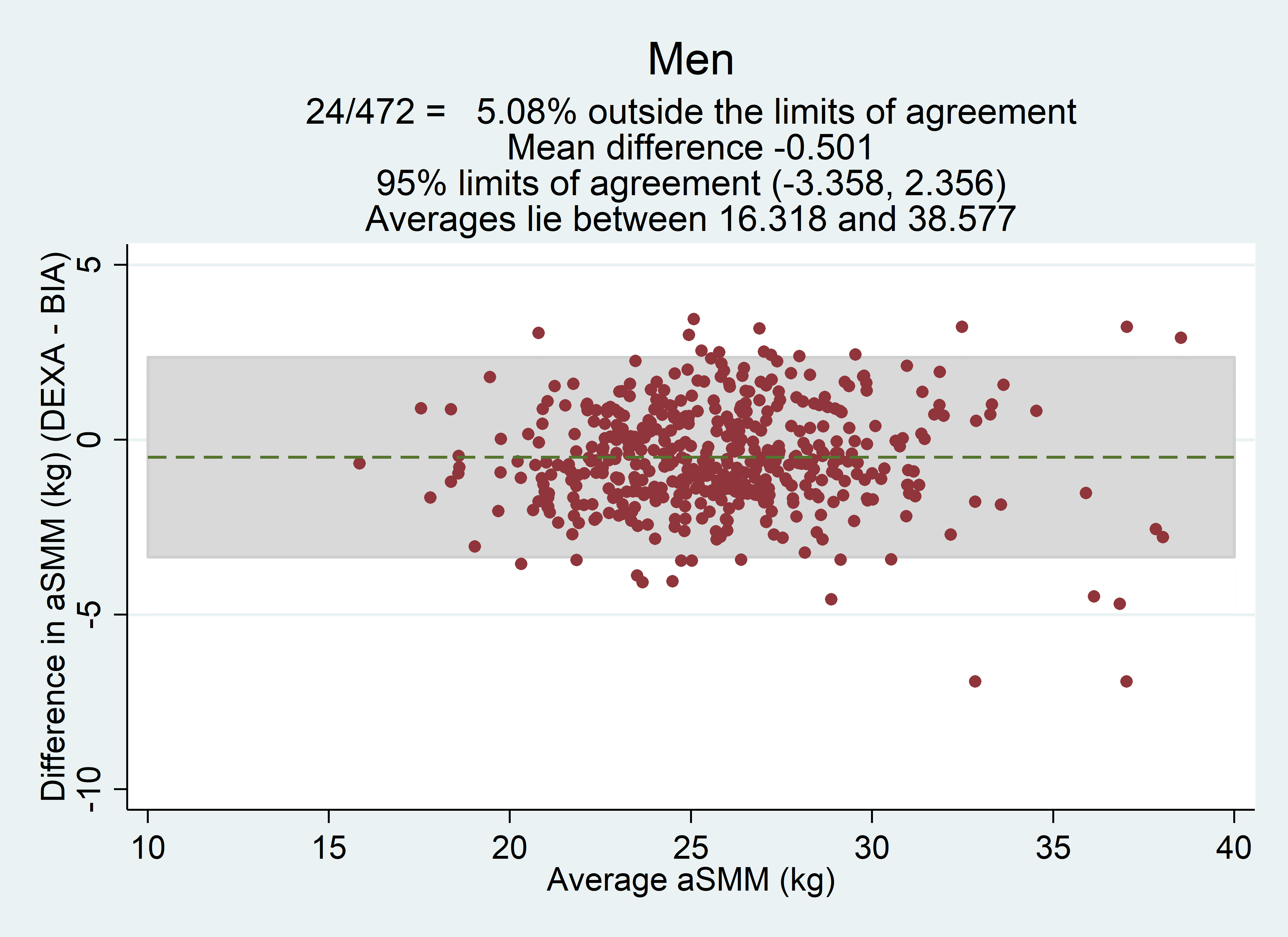

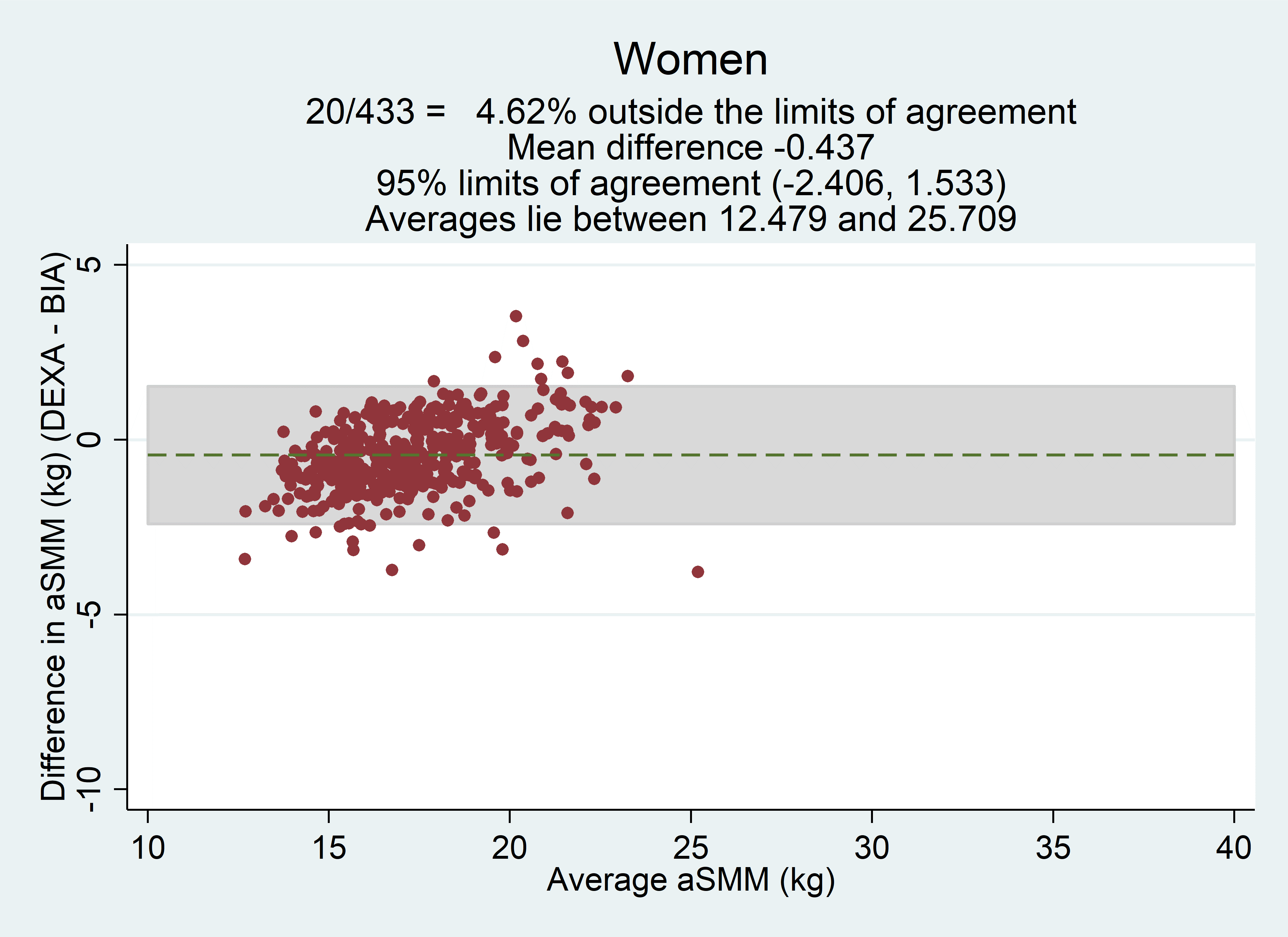
Figure S1 Bland Altman Plots for the agreement between the body composition measurements taken with BIA and DEXA in a subsample of participants.

### Appendix S2 – Modelling the expected trajectories in body composition

**Methods**

The expected trajectories of SMMI and FMI were constructed at discrete time points using data from the participants who came back at the follow-up. Given that participants have only one follow-up, occurring at various intervals, the growth-curve model is not appropriate for constructing expected trajectories. Instead, the construction is based on regression to the mean[23]. Given the z-score at the first time point, $Z_{1}$, the expected z-score at the second time point, $Z_{2}^{\exp}$, is the z-score at the first time point multiplied by the correlation coefficient of the z-scores between the two time point, $\gamma$:

*Equation 1* $Z_{2}^{\exp}=\gamma Z_{1}$

First, the correlation coefficients between any two ages were calculated. This resulted in $30\times29/2=435$ (30 is the number of the ages between 40 and 69) correlation coefficients for each sex with each body composition measure. The correlation coefficients varied considerably for the follow-up interval 2 years and 7 years, so these were excluded from the regression model below.

A regression model was used to smooth the correlation coefficients as function of age and follow-up interval. To do this, the correlation coefficients were first transformed into Fisher’s Z and then regressed on the mean age and the time interval between the two time points to which the correlation coefficient corresponds (Supplemental Tables 6 and 7). The model predicts the Fisher’s Z between any two ages, which were then transformed back to correlation coefficients and used to construct the expected pathway according to Equation 1.

For illustration purposes, the expected trajectories were constructed starting at age 40 year and z-scores between $-2$ and 2, spaced 2/3. The time interval was chosen to be 5 years. That is, Equation 1 was repeated recursively 5 times to obtain the expected z-scores at age 45, 50, 55, 60 and 65. For the ease of visualisation, the resulting expected z-scores were converted back to the measurement scale.

We use the cut off of 5% to define the upper and lower limits of the expected value. The lower limit is $Z_{lower}= \gamma Z_{1}-1.645 \sqrt{1-\gamma^{2}}$ and the upper limit is $Z_{upper}= \gamma Z_{1}+1.645 \sqrt{1-\gamma^{2}}$ , where 1.645 is the critical value for the tail area of 0.05 under the standard normal curve.

1. Stevens, J., Truesdale, K. P., McClain, J. E., & Cai, J. (2006). The definition of weight maintenance. International journal of obesity, 30(3), 391. [↑](#footnote-ref-1)
